# LncRNA‐AK137033 inhibits the osteogenic potential of adipose‐derived stem cells in diabetic osteoporosis by regulating Wnt signaling pathway via DNA methylation

**DOI:** 10.1111/cpr.13174

**Published:** 2021-12-24

**Authors:** Shuanglin Peng, Yujin Gao, Sirong Shi, Dan Zhao, Huayue Cao, Ting Fu, Xiaoxiao Cai, Jingang Xiao

**Affiliations:** ^1^ Department of Oral Implantology The Affiliated Stomatology Hospital of Southwest Medical University Luzhou China; ^2^ State Key Laboratory of Oral Diseases West China Hospital of Stomatology Sichuan University Chengdu China; ^3^ National Key Clinical Specialty The Affiliated Hospital of Southwest Medical University Luzhou China; ^4^ Orofacial Reconstruction and Regeneration Laboratory The Affiliated Stomatology Hospital of Southwest Medical University Luzhou China; ^5^ Department of Oral and Maxillofacial Surgery The Affiliated Stomatology Hospital of Southwest Medical University Luzhou China

**Keywords:** adipose‐derived stem cells, AK137033, diabetic osteoporosis, DNA methylation, osteogenic potential, Wnt signaling pathway

## Abstract

**Objectives:**

Bone tissue engineering based on adipose‐derived stem cells (ASCs) is expected to become a new treatment for diabetic osteoporosis (DOP) patients with bone defects. However, compared with control ASCs (CON‐ASCs), osteogenic potential of DOP‐ASCs is decreased, which increased the difficulty of bone reconstruction in DOP patients. Moreover, the cause of the poor osteogenesis of ASCs in a hyperglycemic microenvironment has not been elucidated. Therefore, this study explored the molecular mechanism of the decline in the osteogenic potential of DOP‐ASCs from the perspective of epigenetics to provide a possible therapeutic target for bone repair in DOP patients with bone defects.

**Materials and methods:**

An animal model of DOP was established in mice. CON‐ASCs and DOP‐ASCs were isolated from CON and DOP mice, respectively. *AK137033* small interfering RNA (SiRNA) and an *AK137033* overexpression plasmid were used to regulate the expression of *AK137033* in CON‐ASCs and DOP‐ASCs in vitro. Lentiviruses that carried shRNA‐AK137033 or *AK137033* cDNA were used to knockdown or overexpress *AK137033*, respectively, in CON‐ASCs and DOP‐ASCs in vivo. Hematoxylin and eosin (H&E), Masson's, alizarin red, and alkaline phosphatase (ALP) staining, micro‐computed tomography (Micro‐CT), flow cytometry, qPCR, western blotting, immunofluorescence, and bisulfite‐specific PCR (BSP) were used to analyze the functional changes of ASCs.

**Results:**

The DOP mouse model was established successfully. Compared with CON‐ASCs, *AK137033* expression, the DNA methylation level of the sFrp2 promoter region, Wnt signaling pathway markers, and the osteogenic differentiation potential were decreased in DOP‐ASCs. In vitro experiments showed that *AK137033* silencing inhibited the Wnt signaling pathway and osteogenic ability of CON‐ASCs by reducing the DNA methylation level in the sFrp2 promoter region. Additionally, overexpression of *AK137033* in DOP‐ASCs rescued these changes caused by DOP. Moreover, the same results were obtained in vivo.

**Conclusions:**

LncRNA‐AK137033 inhibits the osteogenic potential of DOP‐ASCs by regulating the Wnt signaling pathway via modulating the DNA methylation level in the sFrp2 promoter region. This study provides an important reference to find new targets for the treatment of bone defects in DOP patients.

## Graphical abstract

In this work, the diabetic osteoporosis (DOP) animal model was successfully constructed. DOP‐ASCs were successfully isolated and cultured from DOP mice. In vitro and in vivo studies have demonstrated that LncRNA‐AK137033 regulates the osteogenic potential of DOP‐ASCs by modulating the Wnt signaling pathway via DNA methylation in the sFrp2 promoter region.

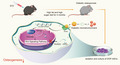

## 1 INTRODUCTION

Diabetes mellitus (DM) is a systemic metabolic disease characterized by hyperglycemia. This systemic glucose metabolism disorder has a serious negative effect on the skeletal system by causing severe complications of the bone and joint system, ie, diabetic osteoporosis (DOP).^1^, ^2^ In addition to the hyperglycemic microenvironment, DOP patients are also characterized by bone microstructure damage, bone strength reduction, fracture susceptibility, and bone defects that are not easily healed.^3^ For bone defects in DOP patients, the current treatment methods are not ideal.^4^, ^5^ With the rapid development of tissue engineering, bone tissue engineering, which includes scaffold materials, seed cells, and growth factors, is considered to be the most promising method for bone defect repair.^6^ Adipose‐derived stem cells (ASCs) are some of the most widely used seed cells in bone tissue engineering. However, our previous studies have shown that, compared with control adipose‐derived stem cells (CON‐ASCs), diabetic osteoporosis adipose‐derived stem cells (DOP‐ASCs) have less osteogenic potential,^7^, ^8^ which limits their application to the treatment of fractures and bone defects in DOP patients. Therefore, the molecular mechanism of the osteogenic decline of DOP‐ASCs requires further exploration to find potential therapeutic targets for the treatment of bone defects in DOP patients.

Wnt signaling pathways are a group of multifunctional signal transduction pathways activated by the binding of the Wnt ligand to the cell membrane receptor. They participate in various physiological and pathological processes of cells, which include various bone tissue diseases such as osteoporosis and stem cell–related bone regeneration.^9^ Activation of the Wnt signaling pathway in stem cells improves their ability of bone differentiation, whereas the inhibition of the Wnt signaling pathway reduces bone formation.^10^, ^11^ Our previous studies have shown that the decrease in the osteogenic potential of DOP‐ASCs compared with CON‐ASCs is related to the Wnt signaling pathway.^8^ However, the molecular mechanism that underlies the regulation of the Wnt pathway for the osteogenic potential of DOP‐ASCs is unclear.

Mammalian DNA methylation refers to methylation of the fifth carbon atom on cytosine in the CpG dinucleotide of DNA, which is catalyzed by four DNA methyltransferases, namely DNMT1, DNMT2, DNMT3a, and DNMT3b. It is generally believed that hypermethylation of DNA is related to the inhibition of gene expression, whereas DNA demethylation has the opposite effect.^12^, ^13^ Because the promoter regions of many genes contain high‐density CpG dinucleotide aggregation regions, namely CpG islands, DNA methylation plays an important role in mammalian cell biology.^14^, ^15^ Recent studies have shown that DNA methylation may affect the multidirectional differentiation of stem cells by regulating the expression of specific genes, which results in various bone diseases that include osteoporosis and osteoarthritis.^16^, ^17^ Therefore, exploring DNA methylation provides a possibility to investigate novel molecular mechanisms of the osteogenic decline in DOP‐ASCs.

Protein coding genes have been studied extensively, but they account for only 1.5% of the human genome.^18^ Noncoding RNAs (ncRNAs), which account for up to 98% of the genome, were once ignored. These ncRNAs are divided into long noncoding RNAs (LncRNAs) and short‐chain ncRNAs in accordance with the transcript length. LncRNAs are a class of ncRNAs with transcript lengths longer than 200 bases, which are localized in the nucleus or cytoplasm.^19^, ^20^ With the gradual deepening of the research on LncRNAs, many studies have reported that LncRNAs regulate gene expression at the epigenetic, transcriptional, and post‐transcriptional levels, and have a potential regulatory effect on the cell fate of mesenchymal stem cells and the occurrence and development of specific diseases.^19^, ^21^, ^22^ In particular, studies have confirmed that LncRNAs affect the expression of specific genes by regulating the DNA methylation level in their promoter region.^23^, ^24^, ^25^ However, the molecular mechanism of LncRNAs in regulating the osteogenic differentiation and bone regeneration of DOP‐ASCs is unclear. Therefore, an epigenetic mechanism, such as the regulation of LncRNAs or DNA methylation in the osteogenic ability of DOP‐ASCs, has become a possible explanation for the decline in the osteogenic potential of DOP‐ASCs.

In the present study, we subjected CON‐ASCs and DOP‐ASCs to mRNA/LncRNA expression profiling and MeDIP sequencing. The results showed a significant difference in the DNA methylation level of the promoter region of the Wnt signaling molecule sFrp2, which was related to LncRNA‐AK137033. Subsequently, we conducted functional studies of sFrp2 and LncRNA‐AK137033 in vivo and in vitro to explore the molecular mechanism that regulates bone differentiation of DOP‐ASCs from the perspective of epigenetics.

## 2 MATERIALS AND METHODS

### 2.1 Diabetic osteoporosis animal model

All procedures related to animal experiments were reviewed and approved by the Ethics Committee of Southwest Medical University (20180391222) and carried out in accordance with the guidelines of the Care and Use of Laboratory Animals (Ministry of Science and Technology of China, 2006). Four‐week‐old male C57BL/6 mice were purchased from the Experimental Animal Center of Southwest Medical University. Fifty mice were randomly divided into CON and DOP groups. The CON group was fed an ordinary diet, and the DOP group was fed a high fat and sugar (HFS) diet. The HFS diet consisted of 65% standard chow, 10% fat, 20% sucrose, 2.5% cholesterol, and other necessary additives (BIOG, Beijing, China). The weight and blood glucose of mice were measured every week. After 4 weeks, 50 mice were fasted for 12 h, followed by injection of streptozotocin (STZ; Sigma, St Louis, USA) in the DOP group (140 mg/kg) or the same volume of citric acid‐sodium citrate buffer (140 ml/kg) in the CON group. After the injection, CON mice were raised on standard chow and DOP mice were still raised on the HFS diet. Additionally, all mice were fed under appropriate conditions (20–25°C with 65%–80% humidity) with free access to drinking water and food. The blood glucose and body weight of each group of mice were recorded every 3–5 days. After 4 months of feeding, the diabetic osteoporosis model was established. Until the end of the study, the mortality rate of STZ‐induced diabetic osteoporosis mice was 20%–33%.

### 2.2 Hematoxylin and eosin (H&E), and Masson's staining

Samples were fixed in 10% paraformaldehyde for 24 h, decalcified in a decalcification solution for about 1 month (this step was omitted for soft tissue), washed with tap water for 48 h, dehydrated with an alcohol gradient, embedded in paraffin, and sectioned. The samples were then stained with H&E and Masson's dye.

### 2.3 Micro‐CT analysis

Samples were fixed with 10% paraformaldehyde for 24 h and then analyzed by a SCANCO Medical CT‐40 (SCANCO Medical, Bassersdorf, Switzerland). Scanning parameters were as follows: voltage, 80 kV; current, 500 μA; exposure time, 200 ms; rotation angle, 220°; CT reconstruction method; and COBRA‐filtered back‐projection reconstruction.

### 2.4 Isolation and culture of CON‐ASCs and DOP‐ASCs

CON‐ASCs and DOP‐ASCs were obtained from inguinal subcutaneous adipose tissue of CON and DOP mice, respectively. An adipose block from the groin was cut into pieces of about 1 mm^3^ and evenly spread in a T25 culture flask. After covering the cap tightly, the flask was turned upside down and incubated at 37°C for about 5 min. Then, α‐modified Eagle's medium (Hyclone, Pittsburgh, USA) with 10% FBS (Schaumburg, USA) was carefully added to the culture flask to completely immerse the tissue blocks. Subsequently, the tissue blocks were incubated at 37°C with 5% CO_2_ for 7–10 days to obtain passage 0 cells. The cells were passaged at 80%–90% confluence, and passage 3 cells were used in experiments.

### 2.5 Characterization of isolated ASCs by flow cytometry

Passage 3 ASCs was resuspended with PBS and stained for CD29, CD44, CD45, CD90, CD31, or CD34 with fluorophore‐conjugated antibodies at 4°C for 30 min. Unstained cells were used as a blank control. After washing with PBS, the cells were analyzed using a FACS Calibur flow cytometer (BD Biosciences, San Jose, USA).

### 2.6 Quantitative polymerase chain reaction

Quantitative polymerase chain reaction (qPCR) was used to measure the mRNA expression levels of *AK137033*, secreted frizzle‐related protein 2 (*sFrp2*), cadherin‐associated protein, delta 1 (*β*‐*catenin*), osteopontin (*Opn*), and runt‐related transcription factor 2 (*Runx2*) in ASCs after osteogenic induction. Primer sequences are presented in Table 1. Total RNA from ASCs was extracted using an RNeasy Plus Mini kit (Qiagen, Hilden, Germany) in accordance with the manufacturer's instructions, and a PrimeScript RT reagent kit with gDNA Eraser (Takara Bio, Tokyo, Japan) was then used to synthesize cDNA from the total RNA. qPCR was performed using a SYBR Premix ExTaq kit (Takara Bio, Tokyo, Japan) and ABI 7300 system (Applied Biosystems, Wilmington, USA) in accordance with the manufacturers’ instructions with the following parameters: 95°C for 30 s and then 45 cycles of 95°C for 5 s and 60°C for 34 s. Glyceraldehyde phosphate dehydrogenase (GAPDH) was used as the internal reference.^26^

**TABLE 1:** Primer sequences for qPCR amplification of specific genes

Genes	Sequence (5’→3’)
*Gapdh*	
Forward	GGTGAAGGTCGGTGTGAACG
Reverse	CTCGCTCCTGGAAGATGGTG
*AK137033*	
Forward	GCATGTACCCCACATTTCAGC
Reverse	CCAGCAATACACAGCAGGAC
*sFrp2*	
Forward	CGTGGGCTCTTCCTCTTCG
Reverse	ATGTTCTGGTACTCGATGCCG
*β‐Catenin*	
Forward	GCTGCGTGGACAATGGCTACTC
Reverse	AGCGTCAAACTGCGTGGATGG
*Opn*	
Forward	TCCCTCCCGGTGAAAGTGACTG
Reverse	TCCTCGCTCTCTGCATGGTCTC
*Runx2*	
Forward	GACTGTGGTTACCGTCATGGC
Reverse	ACTTGGTTTTTCATAACAGCGGA

### 2.7 Western blot analysis

Protein expression levels in ASCs after osteogenic induction were measured by western blotting. Total protein was extracted from ASCs using a total protein extraction kit (Keygen Biotech, Nanjing, China) in accordance with the manufacturer's instructions. Protein samples were mixed with loading buffer and boiled for 5 min for denaturation. After separation by SDS‐PAGE, the protein samples were transferred to a polyvinylidene fluoride membrane by the wet transfer method and blocked in 5% dry skim milk at 37°C for 1 h. The blots were then incubated with diluted primary antibodies against sFrp2 (abs134753) (Absin, Shanghai, China), GSK‐3β (12456), p‐GSK‐3β (5558) (Cell Signaling Technology, Danvers, USA), GAPDH (ab181602), β‐catenin (ab32572), OPN (ab8448), or RUNX2 (ab92336) (Abcam, Cambridge, UK) with shaking at 4°C overnight. The following day, the membranes were washed three times with Tris‐buffered saline with Tween‐20 (TBST) and then incubated with diluted HRP‐labeled goat anti‐rabbit or anti‐mouse secondary antibodies for 1 h. Then, the membranes were washed three times with TBST and developed with an enhanced chemiluminescence detection system (Bio‐Rad, Hercules, USA).^27^, ^28^

### 2.8 Immunofluorescence staining

Immunofluorescence staining was used to analyze the relative expression of RUNX2 and OPN protein in ASCs. CON‐ASCs and DOP‐ASCs after osteogenic induction were washed with PBS for three times, fixed with 4% paraformaldehyde for 30 min, and then permeabilized with 0.5% Triton X‐100 for 10 min. Subsequently, the samples were incubated with 5% sheep serum at 37℃ for 1 h and then incubated with diluted primary antibodies (anit‐RUNX2 or ‐OPN) overnight. The following day, the samples were rewarmed at 37°C for 1 h and then incubated with a diluted fluorescent dye‐conjugated anti‐rabbit secondary antibody (Invitrogen, CA, USA) at 37°C for 1 h. The cytoskeleton and nucleus of ASCs were stained with phalloidin and 4′6‐diamidino‐2‐phenylindole, respectively. Fluorescence images of each sample were obtained under a confocal laser microscope (Nikon, Tokyo, Japan).^29^, ^30^

### 2.9 Cell transfection

*AK137033*‐specific SiRNA was synthesized by GenePharma (Shanghai, China), SiRNA sequences are shown in Table 2.

**TABLE 2:** SiRNA sequences designed for *AK137033*

	Sequence (5’→3’)
SiRNA	
Sense	GCAUCAUGCAAUGAGGAAUTT
Antisense	AUUCCUCAUUGCAUGAUGCTT
SiRNA‐NC	
Sense	UUCUCCGAACGUGUCACGUTT
Antisense	ACGUGACACGUUCGGAGAATT

For *AK137033* overexpression, the *AK137033* cDNA sequence was amplified and subcloned into a pcDNA3.1 vector. An empty pGFP3.1 vector that carried eGFP was used as the negative control.

SiRNA and the plasmid were transfected into ASCs using the Auto Electroporator system (Bimake, TX, USA) in accordance with the manufacturer's instructions for in vitro experiments.

For *AK137033*‐specific overexpression, lentivirus and shRNA lentivirus were synthesized by Obio Technology (Shanghai, China). For *AK137033* overexpression, the *AK137033* cNDA sequence was amplified and subcloned into the pSLenti‐EF1‐EGFP‐F2A‐Puro‐WPRE2‐CMV‐MCS lentiviral vector. For *AK137033* knockdown, oligonucleotides with *AK137033* splice variant RNA interference targets were annealed and ligated into the pSLenti‐U6‐shRNA‐CMV‐EGFP‐F2A‐Puro‐WPRE lentiviral vector. The oligonucleotide sequences of shRNA with *AK137033* RNA interference targets are shown in Table 3.

**TABLE 3:** Oligonucleotide sequences of shRNAs with *AK137033* RNA interference targets

	5’	STEM	Loop	STEM	3’
sh‐AK137033‐F	Ccgg	GGTCAACTACTACGTATAT	TTCAAGAGA	ATATACGTAGTAGTTGACC	TTTTTTg
sh‐AK137033‐R	aattcaaaaaa	GGTCAACTACTACGTATAT	TCTCTTGAA	ATATACGTAGTAGTTGACC	
sh‐NC‐F	Ccgg	CCTAAGGTTAAGTCGCCCTCG	CTCGAG	CGAGGGCGACTTAACCTTAGG	TTTTTTg
sh‐NC‐R	aattcaaaaaa	CCTAAGGTTAAGTCGCCCTCG	CTCGAG	CGAGGGCGACTTAACCTTAGG	

AK137033 overexpression or AK137033 knockdown lentiviruses were transfected into ASCs in accordance with the manufacturer's instructions for in vivo experiments.

### 2.10 Alizarin red and alkaline phosphatase (ALP) staining

After 3 or 5 days of osteoinduction, ASCs were rinsed with PBS three times and then fixed with 4% paraformaldehyde at 4°C for 30 min. ALP activity was detected by an Alkaline Phosphatase Assay Kit (Beyotime, Shanghai, China) in accordance with the manufacturer's guidelines. Similarly, after 14 days of osteoinduction, ASCs were rinsed with PBS for three times and then fixed with 4% paraformaldehyde at 4°C for 30 min. Fixed ASCs were incubated in a 0.1% alizarin red solution for 30 min at 37°C to assess the formation of calcium nodules.

### 2.11 Preparation of ASC‐seeded BCP scaffolds

Biphasic calcium phosphate (BCP) scaffolds were provided by Sichuan University Research Center. After autoclaving the scaffolds, ASCs infected with lentiviruses were seeded on BCP scaffolds at 2 × 10^5^/cm^2^ and cultured in osteogenic induction medium for 48 h for subsequent in vivo experiments.

### 2.12 Establishment of a critical‐sized calvarial bone defect model in mice and implantation of ASC‐seeded BCP scaffolds in vivo

ASC‐seeded BCP scaffolds were prepared as the following groups: BCP scaffold seeded with CON‐ASCs (CON‐B), BCP scaffold seeded with CON‐ASCs transfected with the knockdown lentiviral vector (CON‐NC), BCP scaffold seeded with CON‐ASCs with *AK137033* knockdown (CON‐shRNA), BCP scaffold seeded with DOP‐ASCs (DOP‐B), BCP scaffold seeded with DOP‐ASCs infected with the overexpression lentiviral vector (DOP‐NC), and BCP scaffold seeded with DOP‐ASCs overexpressed *AK137033* (DOP‐LVRNA). Fifteen CON mice were randomly divided into three groups, and fifteen DOP mice were randomly divided into three groups. A circular defect of 4 mm in diameter was made on the right calvarium of each mouse under sterile conditions. The prepared scaffolds were implanted into the bone defects (five mice in each group), and then, the muscle layer and skin were sutured. After the operation, the mice were fed under appropriate conditions (20–25°C with 65%–80% humidity).

### 2.13 Statistical analysis

SPSS 19.0 software was used for statistical analysis. Data were tested by the t‐test or one‐way analysis of variance. Each experiment was repeated at least three times. The results are expressed as the mean ± standard deviation (SD). Data were significantly different at *p *< 0.05.

## 3 RESULTS

### 3.1 Successful establishment of the diabetic osteoporosis animal model

**FIGURE 1:**
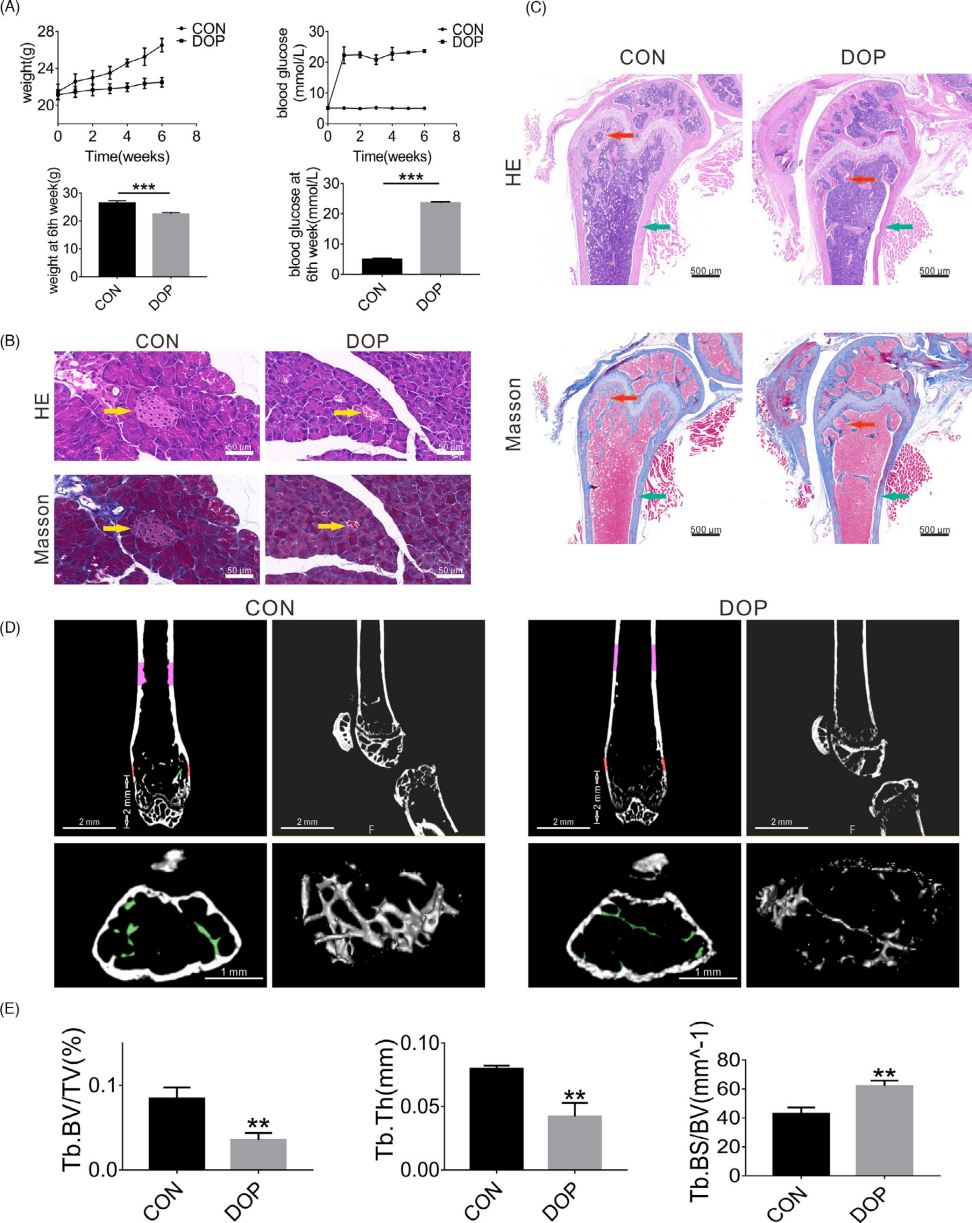
Successful establishment of the diabetic osteoporosis mouse model. A, Compared to CON mice, DOP mice had a lighter body weight and higher blood glucose level (>16.8 M). B, The volume of islet tissue in the DOP group was smaller, vacuolar degeneration had occurred, and inflammatory cells had infiltrated around islets (indicated by yellow arrows). C, D, Histochemical staining and micro‐CT analysis showed that, compared with the femur of the CON group, there was less and disordered bone trabeculae in the DOP group (indicated by red arrows) and the bone cortex had become thinner (indicated by green arrows). E, Statistical analysis of Tb.BV/TV, Tb. Th, and Tb.BS/BV between CON and DOP mice. Data shown as the mean ± SD (*n* ≥ 3), *p < 0.05; **p < 0.01; ***p < 0.001

After STZ injection, we continuously observed changes in body weight and blood glucose in mice. The blood glucose levels of CON mice were <9 M; the blood glucose levels of mice in the DOP group were maintained at >16.8 M; and the weight of mice in the DOP group was lower than that in the CON group (Figure 1A). Compared with the CON group, H&E and Masson's staining of the pancreas showed that the volume of islets tissue in the DOP group was smaller, vacuolar degeneration had occurred, and inflammatory cells had infiltrated around islets (Figure 1B). The femurs of CON and DOP mice were stained with H&E and Masson, and subjected to micro‐CT at 4 months after STZ injection. The results of histochemical staining showed that, compared with the CON group, bone trabeculae were fewer and disordered in the DOP group, and the bone cortex had become thinner (Figure 1C). These results were confirmed by micro‐CT analysis (Figure 1D). Compared with CON mice, statistical analysis showed that Tb.BV/TV and Tb. Th were decreased, but Tb.BS/BV had increased in DOP mice (Figure 1E). On the basis of the above results, we concluded that the DOP mice model was established successfully.

### 3.2 Successful isolation and culture of CON‐ASCs and DOP‐ASCs, and their LncRNA/mRNA expression profiles

**FIGURE 2:**
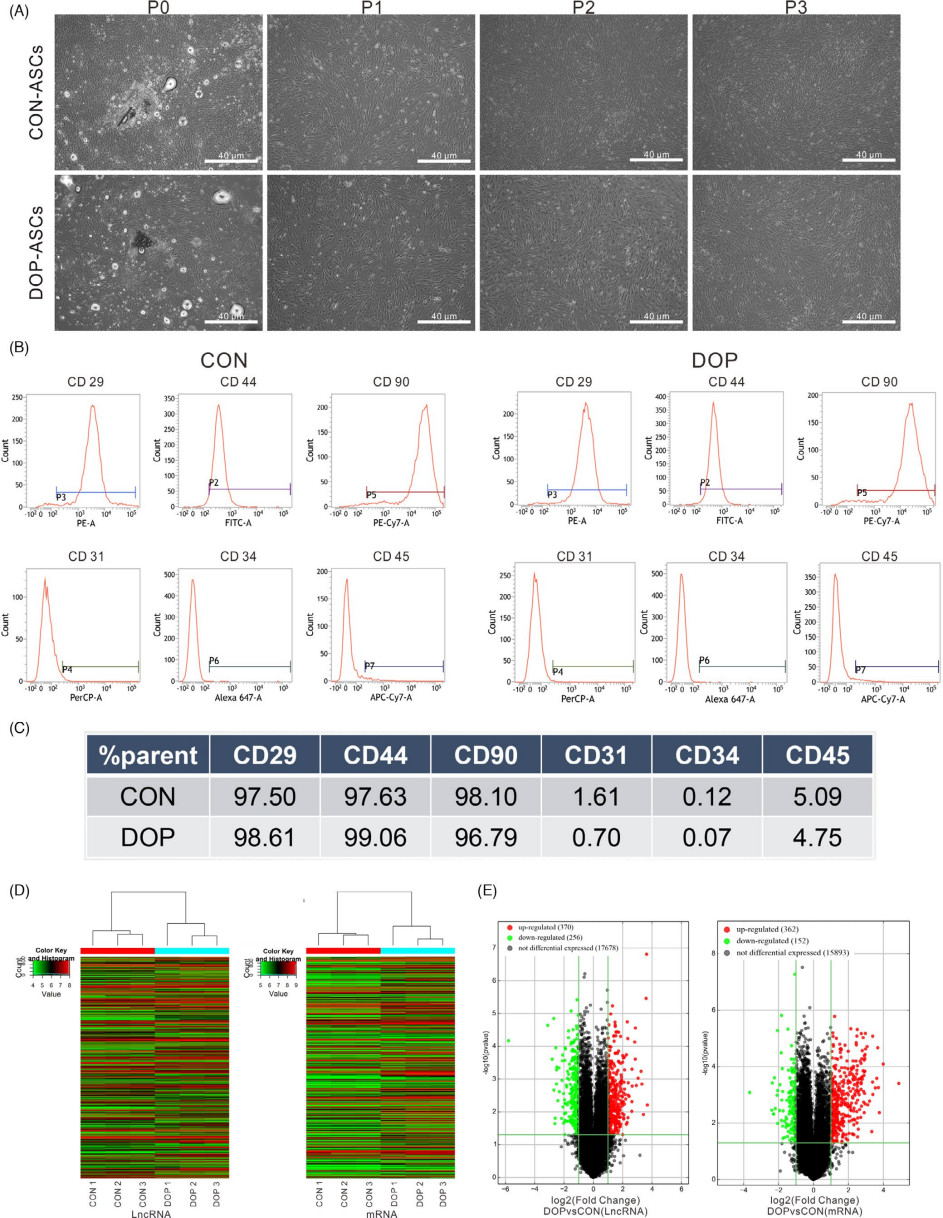
Isolation and culture of CON‐ASCs and DOP‐ASCs, and the results of LncRNA/mRNA expression profiling. A, Normal appearance of CON‐ASCs and DOP‐ASCs observed under an inverted phase contrast microscope. B, C, Surface antigens of passage 3 ASCs detected by flow cytometry. D, E, Cluster and volcano plots of LncRNA/mRNA expression profiles

CON‐ASCs and DOP‐ASCs were obtained from CON mice and DOP mice, respectively. The ASCs of each passage were observed under an inverted phase contrast microscope. Cell culture showed that ASCs grew adherently and were spindle shaped, plump, and distributed evenly (Figure 2A). The surface antigens of the passage 3 ASCs were detected by flow cytometry. Surface antigens CD29, CD44, and CD90 were positive on CON‐ASCs and DOP‐ASCs, and CD31, CD34, and CD45 were negative (Figure 2B, C). These results indicated that the ASCs were highly pure. Compared with CON‐ASCs, cluster and volcano plots of LncRNA/mRNA expression profiles revealed that 370 LncRNAs and 362 mRNAs were upregulated, and 256 LncRNAs and 152 mRNAs were downregulated in DOP‐ASCs (fold change > 1.5, and *p *< 0.05) (Figure 2D, E).

### 3.3 Expression of *AK137033*, the Wnt signaling pathway, and osteogenic differentiation potential are downregulated in DOP‐ASCs

**FIGURE 3:**
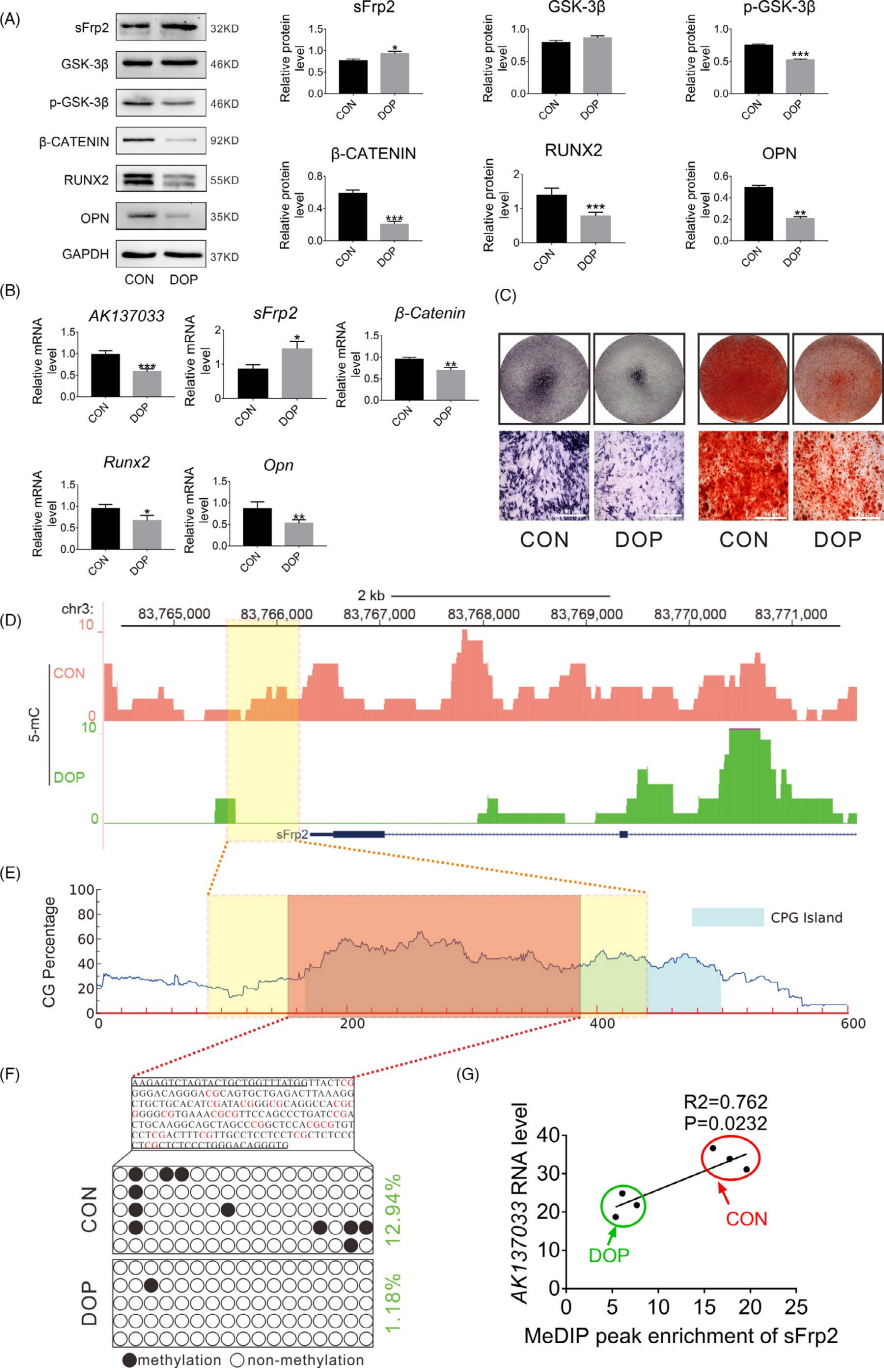
*AK137033* and a change in the methylation levels of the sFrp2 promoter region are involved in the regulation of the osteogenic differentiation potential of DOP‐ASCs. A, Western blot analysis of sFrp2, p‐GSK‐3β, β‐catenin, RUNX2, and OPN in CON‐ASCs and DOP‐ASCs. B, qPCR analysis of *AK137033*, *sFrp2*, *β*‐*Catenin*, *Runx2*, and *Opn* in CON‐ASCs and DOP‐ASCs. C, ALP and alizarin red staining revealed less alkaline phosphatase activity and fewer mineralized nodules in the DOP group than those in the CON group. D, MeDIP sequencing showed that the DNA methylation level of the sFrp2 promoter region in DOP‐ASCs was significantly higher than that in CON‐ASCs (genomic coordinates: chr3, 83765880–83766230). E, Meth Primer analysis showed a large amount of CpG islands (CGIs) in the sFrp2 promoter region (genomic coordinates: chr3, 83765880–83766230). F, BSP confirmed that the methylation level in the sFrp2 promoter region (genomic coordinates: chr3, 83765880–83766230) of CON‐ASCs was higher than that in DOP‐ASCs. G, Correlation analysis of *AK137033* and the DNA methylation level of the sFrp2 promoter region in CON‐ASCs and DOP‐ASCs. Data shown as the mean ± SD (*n* ≥ 3), *p < 0.05; **p < 0.01; ***p < 0.001

After 3 days of osteogenic induction, western blotting was used to measure the protein levels of sFrp2, GSK‐3β, p‐GSK‐3β, β‐catenin, RUNX2, and OPN in CON‐ASCs and DOP‐ASCs. Moreover, the mRNA levels of *AK137033*, *β*‐*Catenin*, *Runx2*, and *Opn* were measured by qPCR. Additionally, ALP and alizarin red staining was used to detect differences in the osteogenic abilities of CON‐ASCs and DOP‐ASCs after 3 and 14 days of osteogenic induction. Compared with CON‐ASCs, the protein and gene expression levels of Wnt signaling pathway makers and osteogenesis‐related molecules, and the expression of LncRNA‐AK137033 were downregulated in DOP‐ASCs (Figure 3A, B). Moreover, ALP and alizarin red staining revealed fewer alkaline phosphatase and mineralized nodules in the DOP group than in the CON group (Figure 3C). These results showed that the expression of *AK137033*, the Wnt signaling pathway, and osteogenic differentiation potential were downregulated in DOP‐ASCs.

### 3.4 Inhibition of the Wnt signaling pathway in DOP‐ASCs may be related to *AK137033* and changes in the DNA methylation level in the sFrp2 promoter region

We performed MeDIP sequencing and mRNA/LncRNA expression profiling of CON‐ASCs and DOP‐ASCs. MeDIP sequencing showed that the DNA methylation peak of the sFrp2 promoter region in CON‐ASCs was significantly higher than that in DOP‐ASCs (genomic coordinates: chr3, 83765880–83766230) (Figure 3D). Interestingly, calculation by Meth Primer software showed a large amount of CpG islands (CGIs) in the sFrp2 promoter region (genomic coordinates: chr3, 83765880–83766230), which is a prerequisite for DNA methylation (Figure 3E). BSP results confirmed that the methylation degree of CON‐ASCs was higher than that of matched DOP‐ASCs in the sFrp2 promoter region (genomic coordinates: chr3, 83765880–83766230) (Figure 3F). Additionally, combined with the results of MeDIP sequencing and mRNA/LncRNA expression profiling, we found a statistical correlation between *AK137033* expression and the methylation level in the sFrp2 promoter region (Figure 3G). These results demonstrated that the inhibition of the Wnt signaling pathway in DOP‐ASCs may be related to *AK137033* and changes in the DNA methylation level in the sFrp2 promoter region.

### 3.5 *AK137033* silencing inhibits the Wnt signaling pathway in CON‐ASCs by reducing the DNA methylation level of the sFrp2 promoter region

**FIGURE 4:**
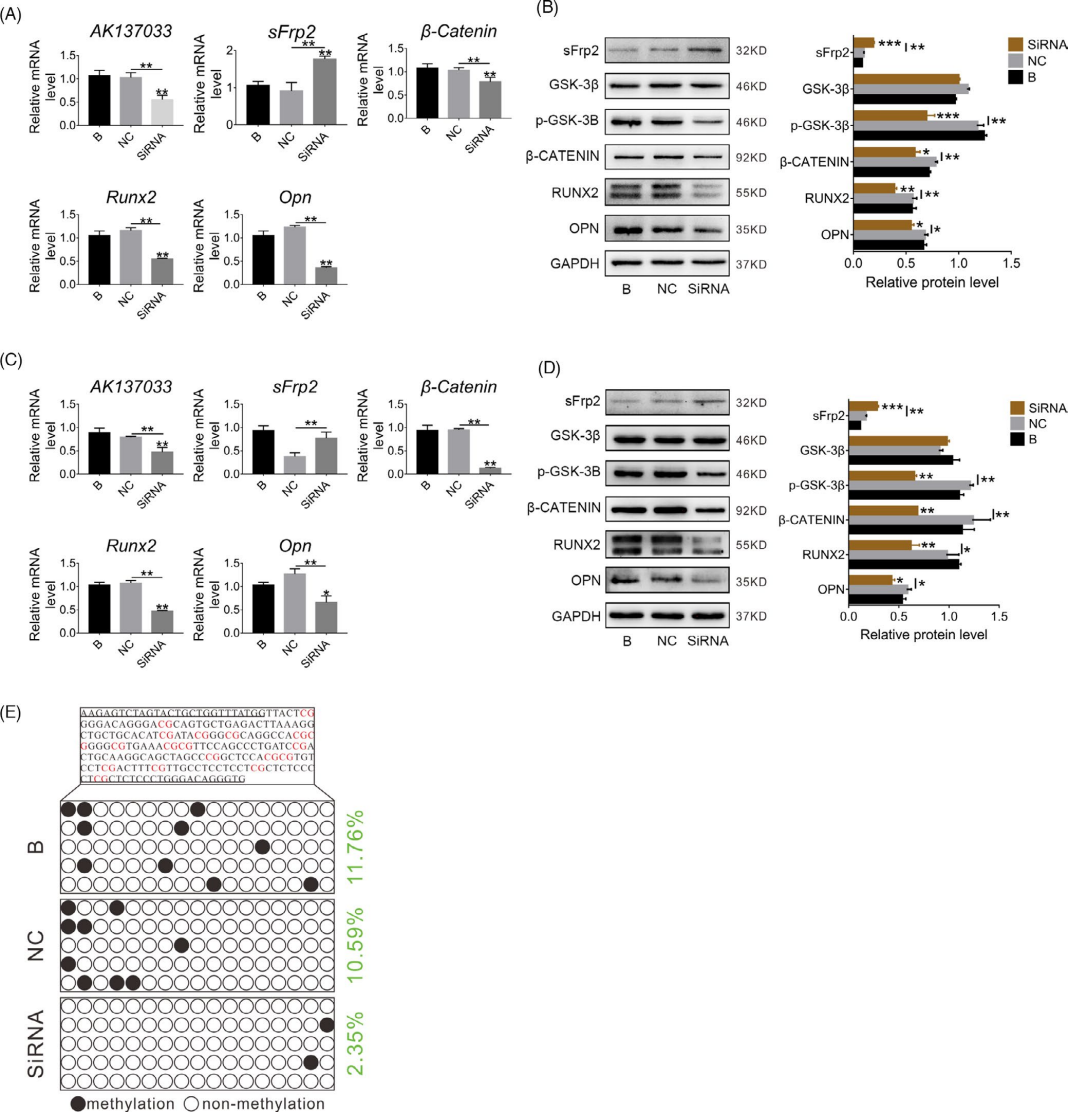
*AK137033* silencing inhibits the Wnt signaling pathway in CON‐ASCs by reducing the DNA methylation level of the sFrp2 promoter region. A, B, The mRNA and protein levels of Wnt signaling pathway makers and osteogenesis‐related molecules in the SiRNA group were decreased after *AK137033* silencing in CON‐ASCs (osteoinduction for 3 days). C, D, After *AK137033* was silenced in CON‐ASCs, the mRNA and protein levels of Wnt signaling pathway markers and osteogenesis‐related molecules were decreased in the SiRNA group (osteoinduction for 6 days). E, BSP results revealed that the DNA methylation level of the sFrp2 promoter region in the SiRNA group was downregulated compared with that in B and NC groups. Data shown as the mean ± SD (*n* ≥ 3), *p < 0.05; **p < 0.01; ***p < 0.001

Previous studies have shown that the inhibition of the Wnt signaling pathway in DOP‐ASCs may be related to *AK137033* and changes in the DNA methylation level in the sFrp2 promoter region, and *AK137033* was highly expressed in CON‐ASCs. Therefore, we silenced *AK137033* by specific siRNAs in CON‐ASCs and then detected the expression of mRNAs and proteins related to the Wnt signaling pathway. CON‐ASCs in the SiRNA group were transfected with si‐*AK137033*, and CON‐ASCs in groups B and NC were treated with ordinary osteogenic induction medium and a siRNA negative control, respectively. At 3 days after osteogenic induction, we performed qPCR and western blot analyses. Compared with B and NC groups, the Wnt signaling pathway markers and downstream osteogenesis‐related molecules in the SiRNA group were suppressed after *AK137033* silencing (Figure 4A, B). Similar results were obtained after 5 days of osteogenesis induction (Figure 4C, D). More importantly, BSP results revealed that the DNA methylation level of the sFrp2 promoter region in the SiRNA group was downregulated after *AK137033* silencing (Figure 4E). These changes indicated that *AK137033* silencing inhibited the Wnt signaling pathway in CON‐ASCs by reducing the DNA methylation level of the sFrp2 promoter region.

### 3.6 *AK137033* silencing decreases the osteogenic ability of CON‐ASCs cells

**FIGURE 5:**
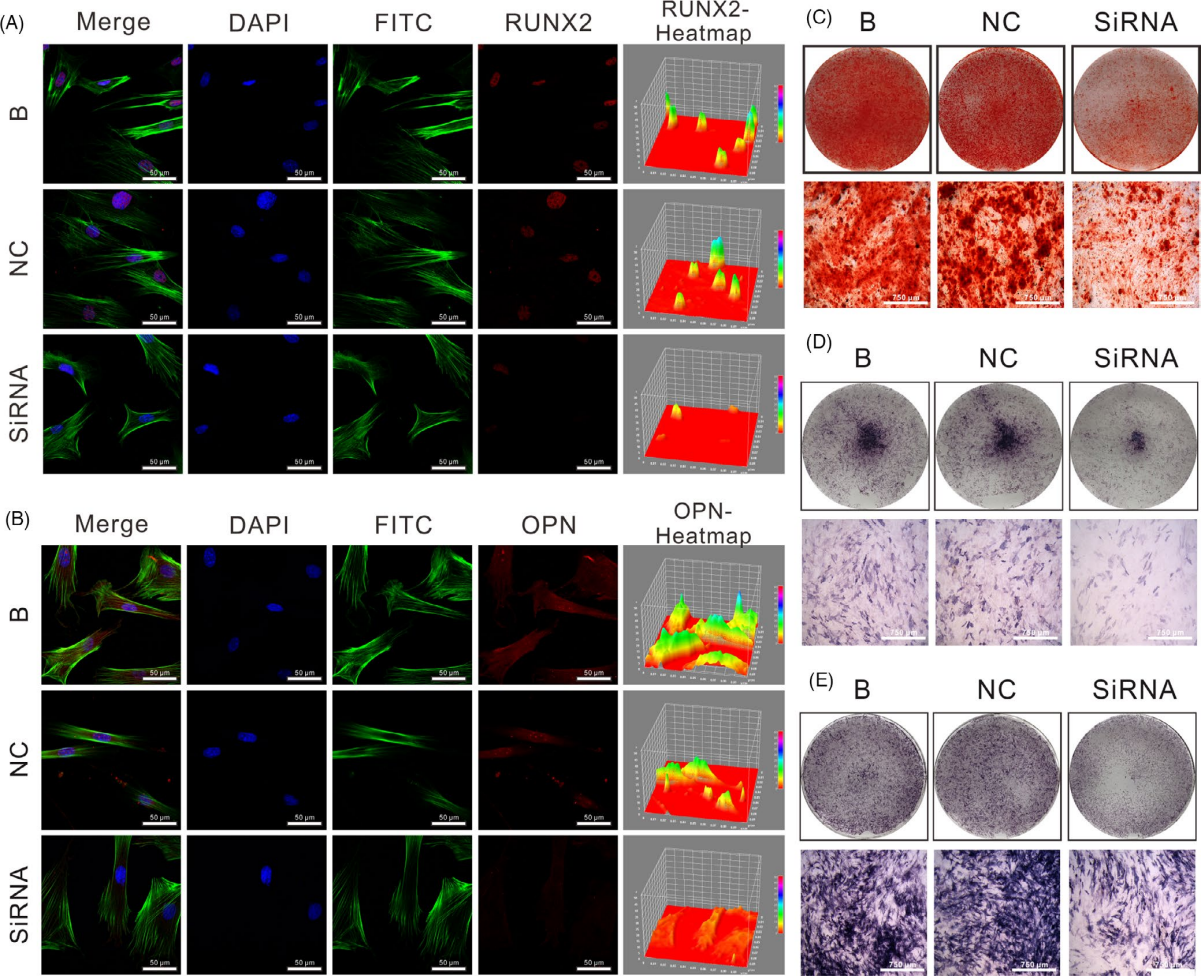
*AK137033* silencing reduces the osteogenic differentiation potential of CON‐ASCs. A, B, At 3 days after osteogenic induction, immunofluorescence staining of RUNX2 and OPN proteins was performed in CON‐ASCs. C, Alizarin red staining showed that the bone differentiation potential of the SiRNA group was decreased compared to that of B and NC groups after 14 days of osteogenesis induction in CON‐ASCs. At 3 (D) or 5 (E) days of osteogenic induction in CON‐ASCs, ALP staining showed that alkaline phosphatase activity of the SiRNA group was lower than that of B and NC groups

To explore changes in the osteogenic differentiation potential after silencing *AK137033* in CON‐ASCs, we performed immunofluorescence, alizarin red, and ALP staining. At 3 days after osteogenic induction, immunofluorescence staining showed that the expression of RUNX2 and OPN in the SiRNA group was decreased compared to that in B and NC groups (Figure 5A, B). Alizarin red staining revealed fewer mineralized nodules in the SiRNA group than in B and NC groups after 14 days of osteogenic induction (Figure 5C). At 3 and 5 days of osteogenic induction, ALP staining showed less alkaline phosphatase produced by the SiRNA group than that by B and NC groups (Figure 5D, E). These results suggested that silencing *AK137033* reduced the osteogenic differentiation potential of CON‐ASCs.

### 3.7 *AK137033* overexpression activates the Wnt signaling pathway in DOP‐ASCs by increasing the DNA methylation level of the sFrp2 promoter region

**FIGURE 6:**
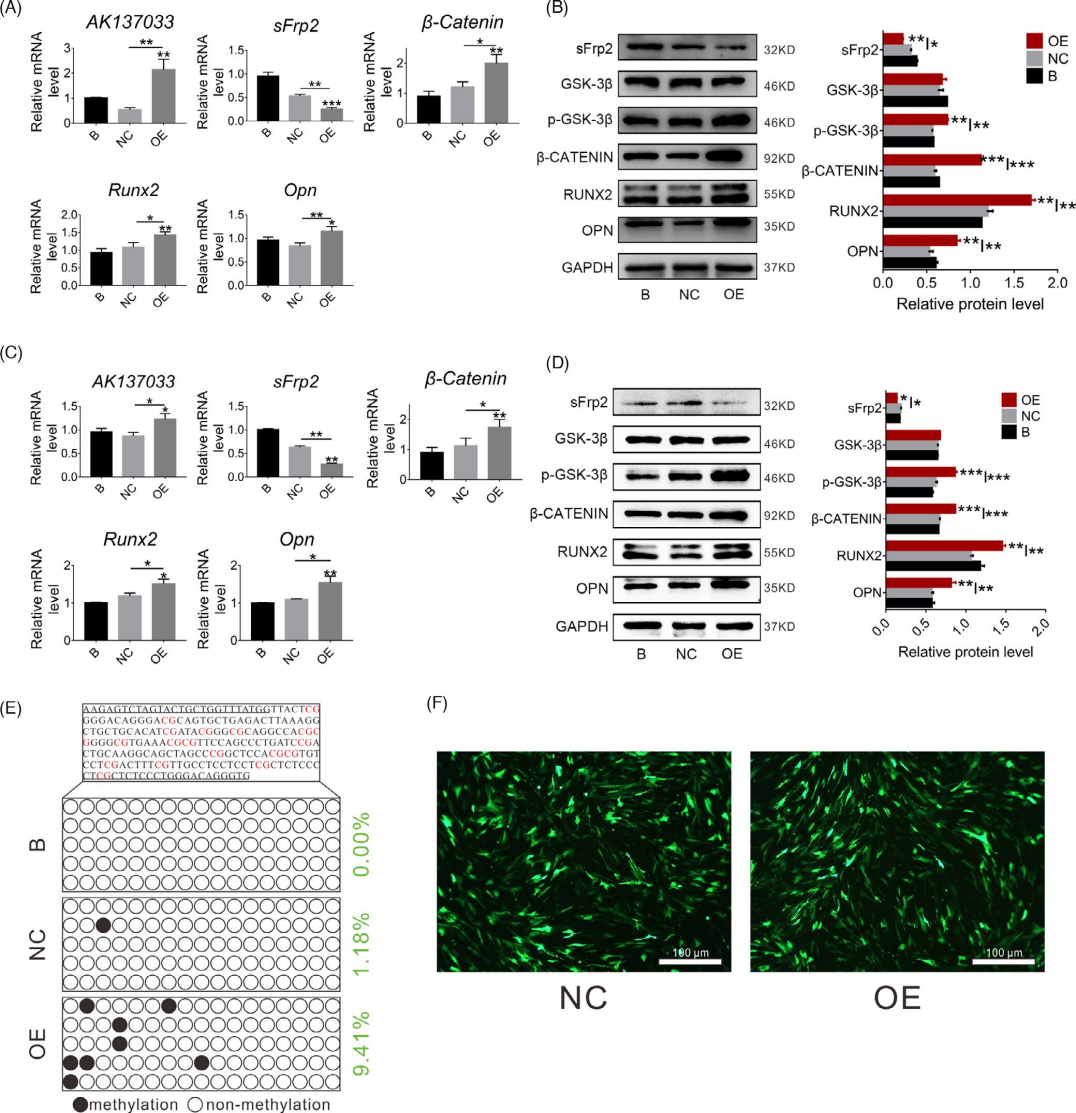
*AK137033* overexpression activates the Wnt signaling pathway in DOP‐ASCs by increasing the DNA methylation level of the sFrp2 promoter region. A, B, The mRNA and protein levels of Wnt signaling pathway makers and osteogenesis‐related molecules in the OE group were increased after *AK137033* overexpression in DOP‐ASCs (osteoinduction for 3 days). C, D, After *AK137033* overexpression in DOP‐ASCs, the mRNA and protein levels of Wnt signaling pathway markers and osteogenesis‐related molecules were increased in the OE group (osteoinduction for 6 days). E, BSP results revealed that the DNA methylation level of the sFrp2 promoter region in the OE group was upregulated compared to that in B and NC groups. F, Cellular uptake of NC and OE plasmids by DOP‐ASCs after treatment for 48 h. Data shown as the mean ± SD (*n* ≥ 3), *p < 0.05; **p < 0.01; ***p < 0.001

At 3 days after osteogenic induction, compared with B and NC groups, qPCR and western blotting showed that the mRNA and protein levels of Wnt signaling pathway markers and downstream osteogenesis‐related molecules were increased in the *AK137033* plasmid (OE) group (Figure 6A, B). Similar results were obtained after 5 days of osteogenic induction (Figure 6C, D). Additionally, BSP results showed that the DNA methylation level of the sFrp2 promoter region in the OE group was increased after *AK137033* overexpression (Figure 6E). To activate the Wnt signaling pathway in DOP‐ASCs, we transfected a plasmid that carried the *AK137033* cDNA sequence into DOP‐ASCs (Figure 6F). Taken together, these observations indicated that *AK137033* overexpression activated the Wnt signaling pathway in DOP‐ASCs by increasing the DNA methylation level of the sFrp2 promoter region.

### 3.8 *AK137033* overexpression increases the osteogenic ability of DOP‐ASCs

**FIGURE 7:**
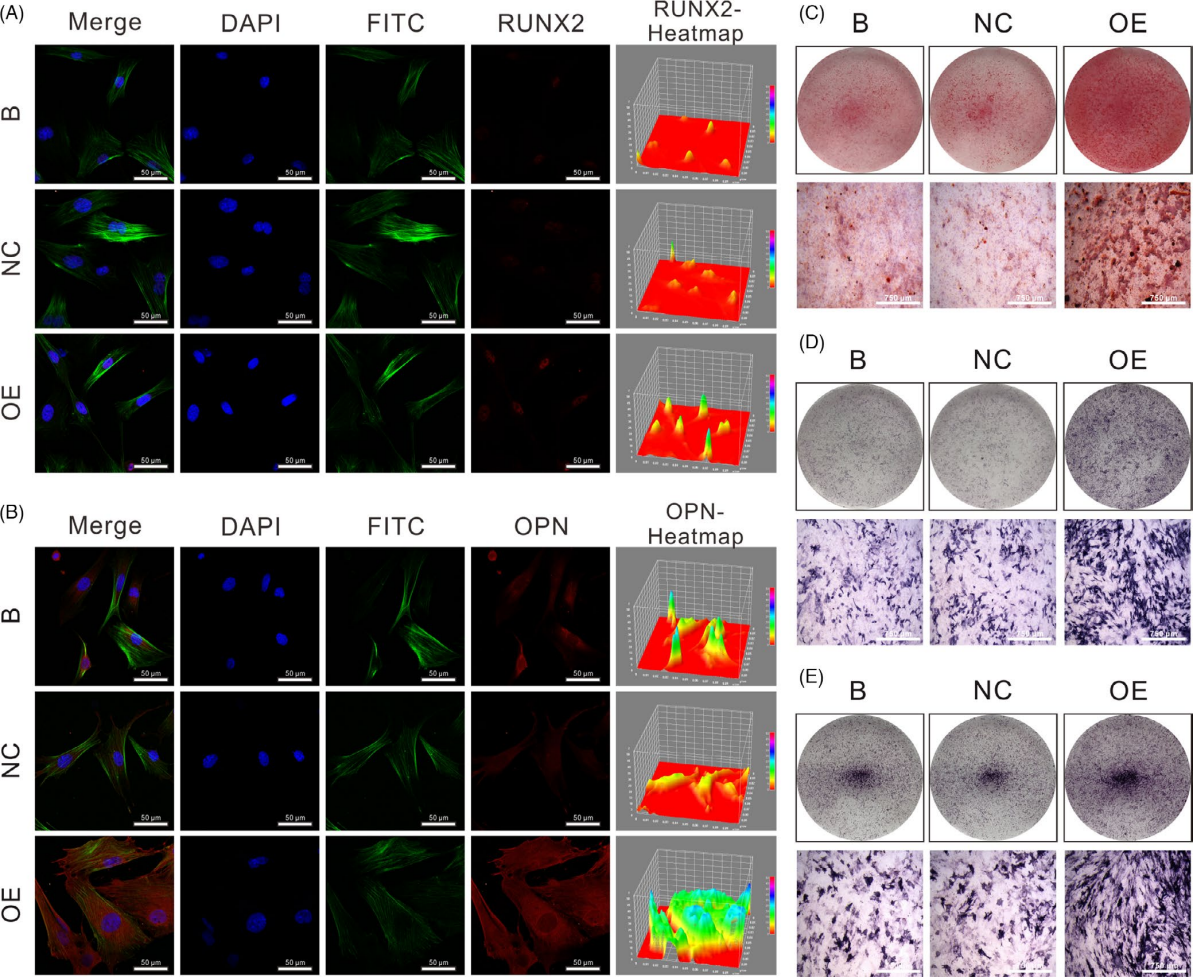
*AK137033* overexpression increases the osteogenic ability of DOP‐ASCs. A, B, At 3 days after osteogenic induction, immunofluorescence staining of RUNX2 and OPN proteins was performed in DOP‐ASCs. C, *AK137033* overexpression rescued the decline of the osteogenic ability of DOP‐ASCs induced by DOP as determined by alizarin red staining (14 days of osteogenic induction). At 3 (D) or 5 (E) days of osteogenic induction in DOP‐ASCs, ALP staining showed that alkaline phosphatase activity in the OE group was higher than that in B and NC groups

To investigate changes in the osteogenic differentiation potential of DOP‐ASCs after overexpression of *AK137033*, immunofluorescence, alizarin red, and ALP staining were performed. After 3 days of osteogenic induction, immunofluorescence staining showed that the expression of RUNX2 and OPN in the OE group was increased compared with that in B and NC groups (Figure 7A, B). Alizarin red staining showed more mineralized nodules in the OE group than those in B and NC groups at 14 days of osteogenesis (Figure 7C). ALP staining revealed higher production of alkaline phosphatase in the OE group than that in B and NC groups at 3 and 5 days of osteogenic induction (Figure 7D, E). These changes indicated that overexpression of *AK137033* enhanced the osteogenic differentiation potential of DOP‐ASCs.

### 3.9 In vivo verification of *AK137033* in regulating the osteogenic ability of ASCs

**FIGURE 8:**
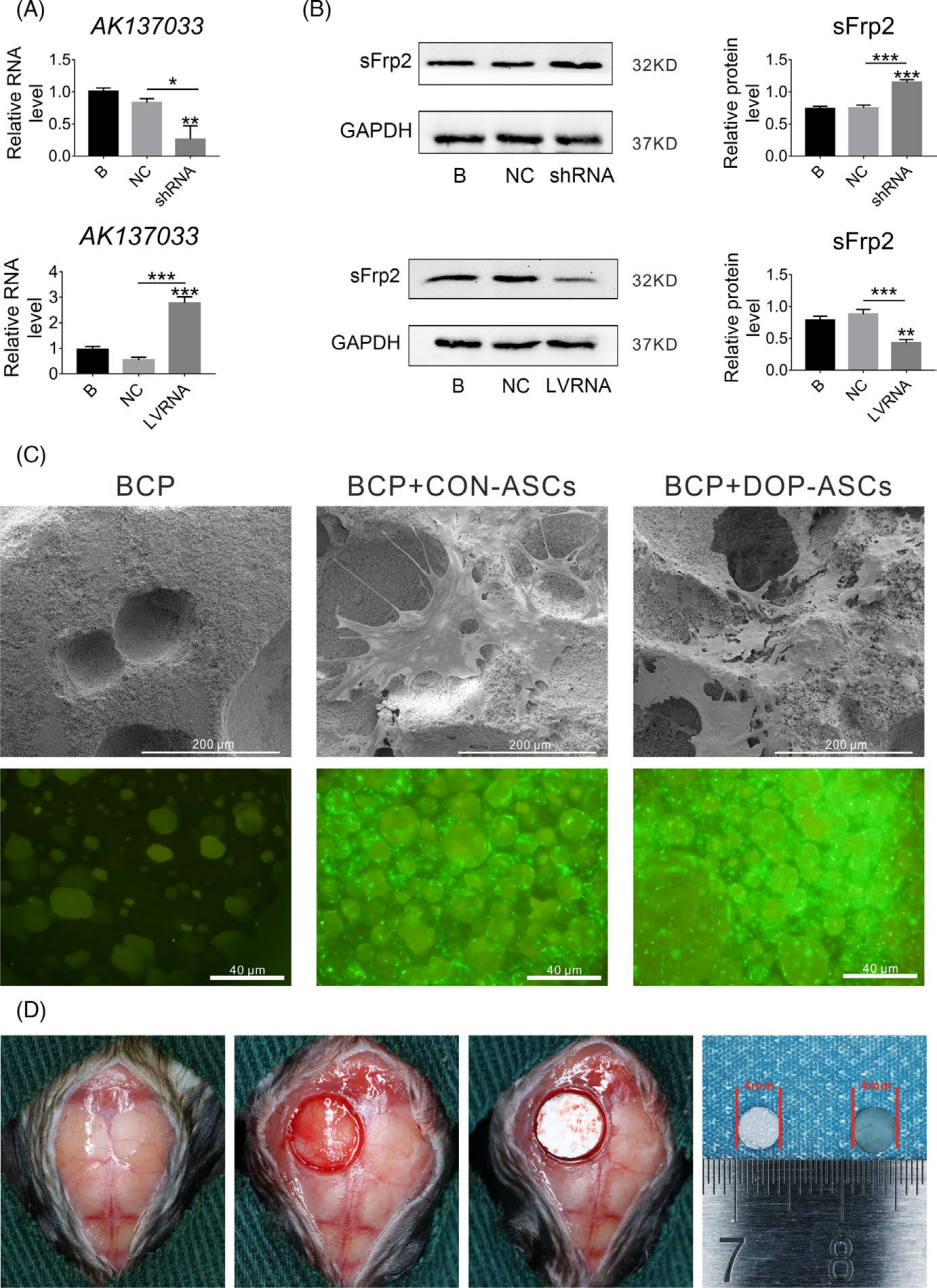
Preparation of ASC‐seeded BCP scaffolds and the critical‐sized calvarial bone defect mouse model. A, B, Transfection efficiency verified by qPCR and western blot analysis after CON‐ASCs were infected with the shRNA lentivirus, and DOP‐ASCs were infected with LVRNA. C, Scanning electron microscopy and fluorescence microscopy of prepared CON‐ASC‐seeded BCP scaffolds and DOP‐ASC‐seeded BCP scaffolds. D, Establishment of the critical‐sized calvarial bone defect mouse model and implantation of ASC‐seeded BCP scaffolds. Data shown as the mean ± SD (*n* ≥ 3), *p < 0.05; **p < 0.01; ***p < 0.001

To further assess the osteogenic role of AK137033 in vivo, we knocked down *AK137033* in CON‐ASCs using a specific shRNA lentivirus (shRNA group) and overexpressed *AK137033* in DOP‐ASCs through a specific LVRNA lentivirus (LVRNA group). After lentivirus infection, the effect was assessed by qPCR and western blotting after 3 days of osteogenic induction. The results showed that the shRNA lentivirus knocked down the expression of *AK137033* in CON‐ASCs and the LVRNA lentivirus overexpressed *AK137033* in DOP‐ASCs (Figure 8A, B). Next, we prepared ASC‐seeded BCP scaffolds. Scanning electron microscopy and fluorescence microscopy showed that CON‐ASCs and DOP‐ASCs adhered to the surface and pores of BCP scaffolds (Figure 8C).

**FIGURE 9:**
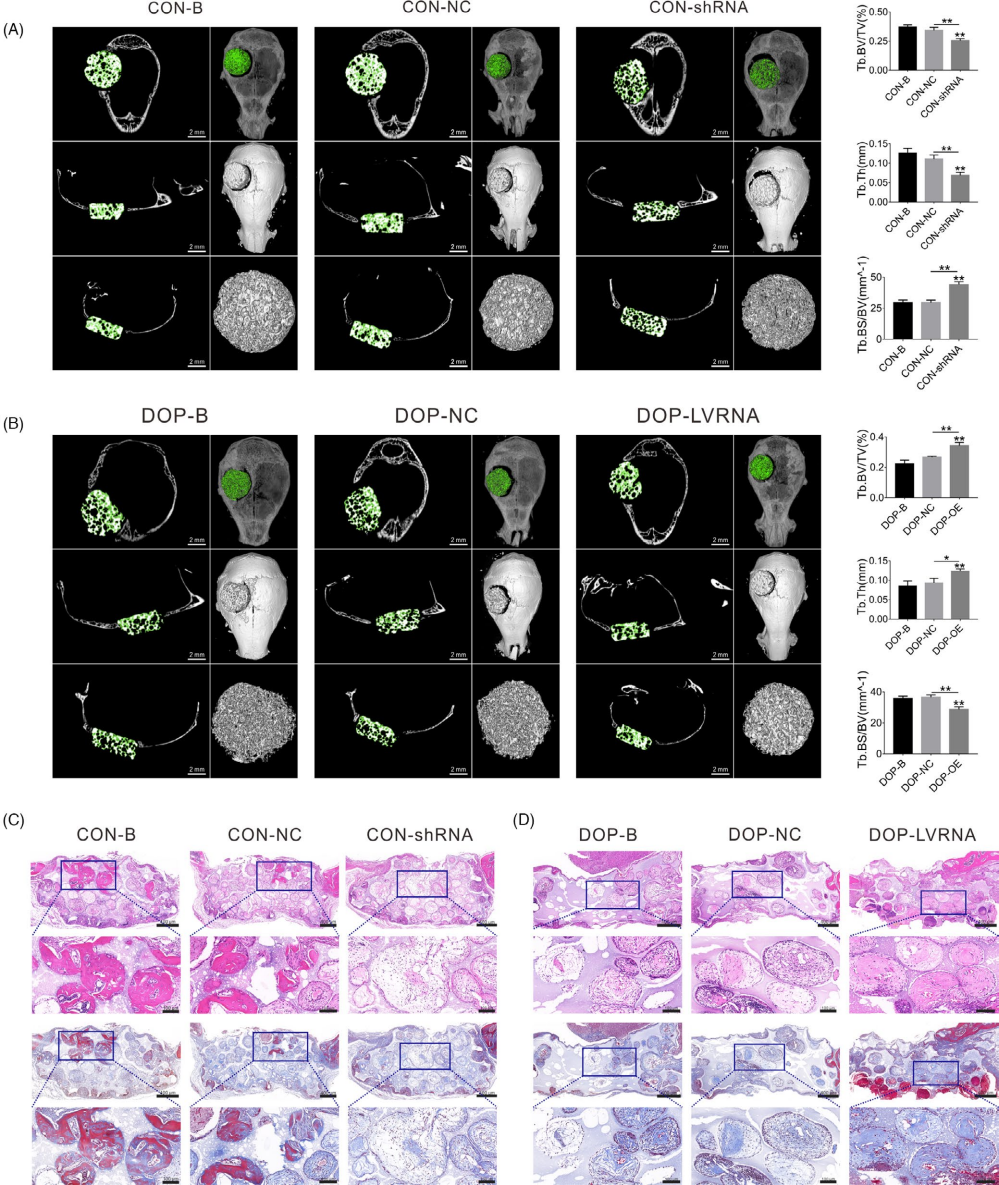
Evaluation of skull repair at 8 weeks after implantation. A, B, Micro‐CT scanning showed that the knockout of *AK137033* decreased Tb.BV/TV and Tb. Th, while Tb.BS/BV was increased in CON mice. In DOP mice, overexpression of *AK137033* reversed the decreases in Tb.BV/TV and Tb. Th, and the increased Tb.BS/BV caused by DOP. C, D, H&E and Masson's staining showed that, in CON mice, the amount of fibrotic and mineralized new bone in the shRNA group was less than that in B and NC groups. In DOP mice, the bone formation ability of the LVRNA group was rescued compared with B and NC groups. Data shown as the mean ± SD (*n* ≥ 3), *p < 0.05; **p < 0.01; ***p < 0.001

Subsequently, we implanted ASC‐seeded BCP scaffolds into a critical‐sized calvarial bone defect model in CON and DOP mice (Figure 8D). Among them, CON mice were implanted with a BCP scaffold seeded with CON‐ASCs (CON‐B), BCP scaffold seeded with CON‐ASCs transfected with the knockdown lentivirus vector (CON‐NC), and BCP scaffold seeded with CON‐ASCs with *AK137033* knockdown (CON‐shRNA); and BCP scaffold seeded with DOP‐ASCs (DOP‐B), BCP scaffold seeded with DOP‐ASCs infected with the overexpression lentivirus vector (DOP‐NC), and BCP scaffold seeded with DOP‐ASCs that overexpressed *AK137033* (DOP‐LVRNA) were implanted in DOP mice. At 8 weeks after transplantation, the formation of mouse calvarial bone was detected by micro‐CT and histochemistry. Micro‐CT scanning showed that the knockout of *AK137033* decreased the bone volume/total volume (Tb.BV/TV) and trabecular thickness (Tb. Th) in CON mice, while the bone surface area/bone volume (Tb.BS/BV) was increased. Conversely, in DOP mice, overexpression of *AK137033* reversed the decrease in bone formation caused by DOP (Figure 9A, B). In CON mice, histochemical staining revealed that the amount of fibrotic and mineralized new bone in the shRNA group was less than that in B and NC groups. In DOP mice, the bone formation ability of the LVRNA group was rescued compared to that in B and NC groups (Figure 9C, D). On the basis of these results, we concluded that *AK137033* regulated osteogenesis of ASCs in vivo. The decrease in the osteogenic differentiation potential of DOP‐ASCs was related to the low expression of *AK137033*.

## 4 DISCUSSION

Diabetes is a group of systemic metabolic diseases characterized by a disturbance in carbohydrate metabolism caused by islet dysfunction or insulin resistance.^31^ Chronic hyperglycemia causes chronic damage to various tissues and organs of the body, which results in various diabetic complications, especially in the eyes, kidneys, blood vessels, and bones.^32^, ^33^, ^34^ Among them, diabetic osteoporosis refers to the metabolic bone disease that occurs because of the hyperglycemic microenvironment.^32^ Recent studies have shown that insulin deficiency or tolerance in diabetic patients leads to disruptions of glucose, lipid, and calcium metabolisms, which results in dysfunctional osteoblasts and osteoclasts and ultimately causes systemic bone loss and a reduction in bone mineral density.^35^, ^36^ Additionally, hyperglycemic microenvironment‐induced autophagy adversely affects the proliferation and differentiation of osteoblasts, which is associated with increases in fracture risk and bone mineral density loss in diabetes.^37^ However, there are few studies on the role of epigenetic alterations of ASCs in the pathogenesis of DOP. The etiology and course of DOP are complex. Therefore, establishment of a DOP animal model is crucial to study the pathogenesis, prevention, and treatment of DOP. STZ injection is considered to be one of the most common methods to establish a DOP animal model.^38^ In this study, we established DOP model mice by injecting STZ. After injection, the DOP group had a steady increase in blood glucose and showed typical diabetic symptoms of increased water and food intake, micturition, and weight loss. Subsequently, the results of histochemical staining and micro‐CT revealed fewer and disordered bone trabeculae in the DOP group compared to those in the CON group, and the bone cortex had become thinner. Therefore, we concluded that the DOP mouse model was established successfully. Next, we isolated and cultured CON‐ASCs and DOP‐ASCs to explore the molecular mechanism by which *AK137033* affects osteogenesis of DOP‐ASCs via DNA methylation.

The Wnt signaling pathway plays an important role in the differentiation and development of stem cells because of high conservation, structural complexity, and the ability of the developmental cascade to integrate the signals of other pathways.^39^ The canonical Wnt pathway (Wnt/β‐catenin) and noncanonical Wnt pathway (Wnt/PCP and Wnt/Ca^2+^) affect bone modeling and reconstruction by regulating the energy metabolism and osteogenesis of osteoblasts.^40^, ^41^, ^42^, ^43^ sFrp2 is an antagonist of the canonical Wnt pathway. It binds to Wnt ligands through a cysteine‐rich domain or C‐terminal netrin‐like domain, or forms nonfunctional complexes with frizzle‐related receptors to inhibit Wnt signaling.^44^ As a crucial molecule of the Wnt signaling pathway, sFrp2 regulates the proliferation, apoptosis, and differentiation of stem cells by inhibiting the Wnt signaling pathway to be involved in multiple biological processes such as cardiac malformations and cardiovascular diseases,^45^ the regulation of skin and hair follicle development,^46^ and the occurrence, development, and prognosis of gastric cancer.^47^ However, no study has revealed the specific role of sFrp2 in regulating the osteogenic potential of DOP‐ASCs through the Wnt signaling pathway. In the present study, we performed mRNA/LncRNA expression profiling and MeDIP sequencing of CON‐ASCs and DOP‐ASCs. The results showed a significant difference in the DNA methylation level of the sFrp2 promoter region in the two groups, which was related to LncRNA‐AK137033. Moreover, we verified the *AK137033* expression level, Wnt signaling pathway difference, and osteogenic differentiation potential of CON‐ASCs and DOP‐ASCs in vitro. The results showed that, compared with CON‐ASCs, the *AK137033* expression level, Wnt signaling pathway, and osteogenic differentiation potential were inhibited in DOP‐ASCs. Moreover, Meth Primer analysis and BSP results demonstrated that the methylation degree of CON‐ASCs was higher than that of matched DOP‐ASCs in the sFrp2 promoter region. The above results supported the mRNA/LncRNA expression profiling and MeDIP sequencing results. However, further functional studies are needed to demonstrate the relationship between the DNA methylation level of the sFrp2 promoter region and *AK137033*.

LncRNAs are a kind of long‐chain RNA (more than 500 nt) that lacks a protein‐coding ability.^48^, ^49^ There is increasing evidence of the roles of LncRNAs in many important biological processes that include gene transcription, mRNA shearing, cell cycle control, epigenetic regulation, and cellular immunity.^50^, ^51^, ^52^ Recent studies have shown that LncRNAs regulate local and distal gene expression at their transcriptional location through multiple mechanisms that include functioning as competing endogenous RNA or acting as monomers with specific domains and recruiting specific DNAs, RNAs, or proteins to regulate downstream gene expression.^53^ DNA methylation refers to modification of specific DNA fragments mediated by DNA methyltransferase families, which results in methylation of the fifth carbon atom on dinucleotide cytosine in CpG islands.^54^, ^55^ DNA methylation in the promoter region inhibits gene expression by recruiting transcription barriers, and DNA methylation in the gene body regulates gene transcription by selective splicing of transcripts or stability of the genome.^56^ As an important part of epigenetics, the association between DNA methylation and LncRNA expression is called LncRNA expression quantitative trait methylation (Lnc‐eQTMs).^57^ A growing number of studies have conducted in‐depth research on Lnc‐eQTMs. Geng et al. found that Lnc‐MAP3K13‐7:1 inhibits the proliferation of ovarian granulosa cells in polycystic ovary syndrome through DNMT1‐mediated hypomethylation of the CDKN1A promoter.^58^ Zheng et al. demonstrated that Lnc‐AK001058 promotes the proliferation, migration, and invasion of colorectal cancer cells by regulating methylation of the ADAMTS12 promoter.^59^ In this study, silencing *AK137033* in CON‐ASCs decreased the DNA methylation level in the sFrp2 promoter region, inhibited the Wnt signaling pathway, and suppressed the osteogenic differentiation potential of CON‐ASCs. Additionally, overexpression of *AK137033* in DOP‐ASCs increased the DNA methylation level in the sFrp2 promoter region, activated the Wnt signaling pathway, and restored the osteogenic differentiation potential of DOP‐ASCs. Interestingly, mRNA/LncRNA expression profiling and MeDIP sequencing of CON‐ASCs and DOP‐ASCs also showed that the difference in the DNA methylation level of the sFrp2 promoter region between the two groups was related to LncRNA‐AK137033. These results suggest that *AK137033* inhibits the osteogenic potential of DOP‐ASCs by regulating the DNA methylation level in the sFrp2 promoter region.

Studies have shown that the diameter of a mouse calvarial bone defect that cannot heal itself is 4 mm.^60^, ^61^ To further assess the osteogenic role of *AK137033* in vivo, a critical‐sized calvarial bone defect model in mice was established and ASC‐seeded BCP scaffolds were implanted. At 8 weeks after transplantation, micro‐CT and histochemistry showed that the knockdown of *AK137033* reduced bone formation in CON mice and overexpression of AK137077 rescued the reduced bone formation ability of DOP mice caused by the hyperglycemic microenvironment. Therefore, we conclude that *AK137033* regulates osteogenesis of ASCs in vivo.

In summary, in vitro and in vivo experiments demonstrated that LncRNA‐AK137033 regulates the osteogenic potential of DOP‐ASCs by modulating the Wnt signaling pathway via DNA methylation in the sFrp2 promoter region. Our study provides an epigenetic explanation for the regulatory mechanism of the osteogenic potential in DOP‐ASCs and is an important reference for the treatment of bone defects in DOP patients.

## CONFLICT OF INTREST

The authors declare that there are no competing interests.

## AUTHOR CONTRIBUTIONS

All authors have made important contributions to this study. Shuanglin Peng conducted in vitro and in vivo experiments, sorted and analyzed the data, and wrote the main manuscript. Yujin Gao established the diabetic osteoporosis mouse model and cultured CON‐ASCs and DOP‐ASCs. Sirong Shi completed mRNA/LncRNA expression profiling, MeDIP sequencing, and in vitro validation experiments. Dan Zhao carried out in vitro experiments and data collection. Huayue Cao and Ting Fu assisted in the in vivo experiments. Xiaoxiao Cai designed the study and revised the manuscript. Jingang Xiao conceived and initiated the study, analyzed the data, and provided funding. All authors have read and approved the manuscript.

## Data Availability

All data included in this article can be obtained from corresponding author upon reasonable requirements.
